# Improvements to the Rapid Detection of the Marine Pathogenic Bacterium, *Vibrio harveyi,* Using Loop-Mediated Isothermal Amplification (LAMP) in Combination with SYBR Green

**DOI:** 10.3390/microorganisms10122346

**Published:** 2022-11-27

**Authors:** Ahmad Mukhlis Abdul Rahman, Julian Ransangan, Vijay Kumar Subbiah

**Affiliations:** 1 Biotechnology Research Institute, Universiti Malaysia Sabah, Jln UMS, Kota Kinabalu 88400, Sabah, Malaysia; 2 Faculty of Chemical Engineering & Technology, Uniciti Alam Campus, Universiti Malaysia Perlis, Sg. Chuchuh, Padang Besar 02100, Perlis, Malaysia; 3 Borneo Marine Research Institute, Universiti Malaysia Sabah, Jln UMS, Kota Kinabalu 88400, Sabah, Malaysia

**Keywords:** rapid detection, loop-mediated isothermal amplification, *Vibrio harveyi*, *toxR* gene, vibriosis, LAMP-SYBR green

## Abstract

The common methods that are presently used to identify *Vibrio harveyi* include microscopic examination and biochemical, immunological and PCR-based assays. These methods require technical expertise, and can be time-consuming. A rapid method is required for the high-throughput screening of large number of samples. As such, we have developed a rapid, simple yet sensitive and specific detection method based on the use of the loop-mediated isothermal amplification (LAMP) of DNA. A set of six primers, i.e., two outer, two inner and two loop primers, was designed based on the in silico analysis of a large pool of 39 strains of the *toxR* gene sequence of *V. harveyi*. The addition of the loop primers decreased the reaction time of the LAMP by more than half. Furthermore, with the application of SYBR Green, the result can be obtained as quickly as in 10 to 15 min without the need of gel electrophoresis. The specificity of the method primers was then determined by performing LAMP with *Vibrio* and non-*Vibrio* samples. LAMP has a greater sensitivity than PCR reaction. The sensitivity of PCR was at 0.6 pg concentration of *V. harveyi* recombinant plasmid DNA standard, while LAMP was able to detect lower amounts even at 0.6 fg. The development of the LAMP assay will provide a valuable tool for the high-throughput rapid detection of *V. harveyi* contamination both in laboratories and in the field.

## 1. Introduction

*Vibrio harveyi* is a gram-negative, rod-shaped, facultative anaerobic microorganism capable of being able to switch between fermentative and respiratory metabolism [1,2]. It occurs naturally in marine habitats, but some strains have developed into significant pathogens of wild and cultured marine fish and invertebrates, especially in warm water. The pathogen is also a zoonosis, as it can infect humans through skin punctures or wounds and may cause severe inflammatory responses [3]. However, *V. harveyi* has found notoriety as a marine pathogen, as it causes severe economic losses for the aquaculture and mariculture industries [4,5]. Diseased fish may exhibit a range of symptoms such as a chronic skin ulcers, gastro-enteritis and deep dermal lesions [6,7,8,9], and it is the main cause of luminous vibriosis in fish, mollusks and crustaceans worldwide [10,11].

Prior to the advent of PCR, the diagnosis of *V. harveyi* was based on the culture in bacterial agar and identification through microscopic examination or biochemical assay [2]. These methods are still important today, particularly when examining the immunological and serological characteristics of the host–pathogen response [12,13]. Nevertheless, the PCR-based approach has become widespread as the definitive technique in the detection of *V. harveyi*. This usually entails the PCR amplification of the 16S rRNA region, the DNA sequencing of the amplicon, and is followed by BLAST analysis for species identification [14,15,16]. Some researchers have developed multiple sets of specific primers for detecting several genes at once in a multiplex PCR approach to increase the specificity of the detection *V. harveyi* and other *Vibrio* species concurrently [17]. Others have used real-time PCR, which offers a more rapid and sensitive approach for the detection of the pathogen compared with conventional PCR [18,19,20]. Here, the detection of amplicons is based on the presence of fluorescent signals (either through the use of fluorescent labeled probes or the intercalating dye SYBR Green) [21].

PCR-based amplification methods (conventional, multiplex or real-time) are usually expensive and time-consuming with the need for specific laboratory equipment, and are not suitable to be used onsite at hatcheries or fish farms that require a high-throughput turnaround time. With this in mind, many researchers have turned to the use of isothermal polymerases, as it does not require the use of the cycling temperatures employed in PCR [22]. Three such methods that are commonly used with isothermal polymerases are loop-mediated isothermal amplification (LAMP) [23], recombinase polymerase amplification (RPA) [24] and rolling circle amplification (RCA) [25]. Of these, LAMP is the more commonly used technique, even though the design of its primers is rather complicated and it requires prior template-sequence information [22]. LAMP was first developed in 2000 for the molecular detection of viruses [26], and it is the method of choice that we have used here. On the other hand, RPA requires substantial protocol optimizations, as the background noise can interfere with the results, and RCA only works with a circular nucleic acid template [22].

The most significant advantage of LAMP is its ability to amplify target DNA under isothermal condition, usually between 60 °C and 65 °C, with high specificity, efficiency and speed. This LAMP method has been tested in the diagnoses of viruses [27,28], bacteria [29], protozoa [30] and fungi [31], and it became popular after 2003 following the emergences of the West Nile and SARS viruses [32,33] and more recently due to SARS-CoV-2 [34,35]. It has also been applied for the rapid detection and identification of aquaculture pathogens [36]. In terms of product visualization, the tube containing products of LAMP can be visualized in the presence of fluorescent intercalating dyes such as SYBR Green. For positive amplification, the color of the dye will change from orange to green when the presence of LAMP products are detected [32,37].

Several papers have previously reported the development of LAMP for *V. harveyi*. Noteworthy among these are Cao and colleagues, who in 2010 were the first to develop LAMP for the detection of *V. harveyi* utilizing the *toxR* gene using two inner and outer primers [38]. This was followed by Caipang et al., who designed the primers for LAMP based on the *dnaJ* gene instead of the *toxR* [39]. Both methods had limitations, as the primer sequences were not conserved for most *V. harveyi* strains. Due to this, others have used the *VhhP2* gene instead as the target for LAMP, as the gene is said to be widely distributed in *V. harveyi* strains from different geographical locations and sources [40,41]. It should be noted that Yu and colleagues [40] targeted the *VhhP2* gene due to the fact that they were developing a triplex LAMP simultaneously along with the amplification of the *toxR* gene from *V. anguillarum* and the collagenase gene of *V. alginolyticus*. However, all of the studies above reported a longer detection time (usually more than 90 min) than the methods that we described here, and may not be practical in cases where immediate point-of-care is required [42]. In 2002, Nagamine et al. developed a method that accelerates the LAMP reaction by using additional primers, termed as loop primers [43]. Loop primers have now been incorporated in many LAMP assay for the detection of pathogens [44], but are not available for the detection of *V. harveyi.*

With this in mind, we attempted to reexamine and improve the LAMP assay by incorporating loop primers that will hybridize the stem-loops structure of DNA and prime strand displacement DNA synthesis, which could help to expedite the reaction for rapid detection. In addition, we designed new sets of inner and outer primers that were based on conserved regions of a comprehensive set of lists of all available *V. harveyi* strains-sequence information of the *toxR* gene in GenBank (www.ncbi.nlm.nih.gov (accessed on 1 February 2020)). The changes made here significantly improved the detection of *V. harveyi*, and may be freely incorporated into existing protocols.

## 2. Materials and Methods

### 2.1. Sampling and Isolation of Genomic DNA

The *Vibrio harveyi* strain VHJR7 was obtained from the Borneo Marine Research Institute, Universiti Malaysia Sabah (UMS), Malaysia. The strain was previously isolated from an infected seabass, *Lates calcarifer* (Bloch), cultured in open net cages in Sabah, Malaysia, during a vibriosis outbreak in February 2008 [45]. The bacterial strain had been previously identified using 16S rRNA and the hemolysin gene through DNA sequencing for species confirmation. Genomic DNA was isolated from the *V. harveyi* strain VHJR7 using the I-Genomic DNA Extraction Kit (Intron Biotechnology, Gyeonggi-do, Republic of Korea).

### 2.2. Primer Design for PCR and LAMP Assays

**Figure 1:**
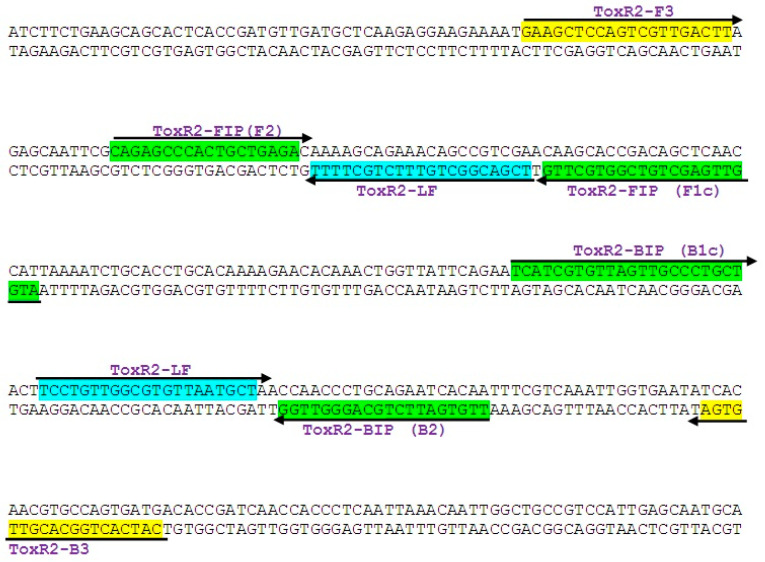
The flanking regions of the inner (green), outer (yellow) and loop (blue) primers based on the *toxR* gene of *V. harveyi* for LAMP reaction.

A set of six newly developed primers for the LAMP assay (Table 1), consisting of two inner primers (*ToxR*2-FIP and *ToxR*2-BIP), two outer primers (*ToxR*2-F3 and *ToxR*2-B3) and two loop primers (*ToxR*2-LF and *ToxR*-LB) were designed according to the published sequences of the *toxR* gene of *Vibrio harveyi* (GenBank ID: DQ640259.1). The primers were designed using the default parameters of Primer Explorer Version 4 at http://primerexplorer.jp/elamp4.0.0/index.html (accessed on 1 February 2020) (Figure 1).

We ensured that the primers were conserved to target all 39 *V. harveyi* strains found in Genbank (Appendix A Table A1). We also performed in silico verification to ensure that the primers were specific to *V. harveyi* and not to any other species under the same genera. The outer primers (*ToxR*2-F3 and *ToxR*2-B3) were used to obtain the PCR amplicon, which was sequenced for validation and subsequently used to produce the template plasmid DNA.

### 2.3. Preparation of DNA Template

The target site of the *V. harveyi toxR* gene (245 bp) was amplified by PCR with approximately 80 ng of *V. harveyi* genomic DNA using the *ToxR*2-F3 and *ToxR*2-B3 primers. The PCR reaction was performed as described previously, with some modifications [46]. The PCR was conducted in a 25 µL total reaction consisting of 1x PCR buffer, 0.2 mM of dNTP mix (Promega, USA), 10 pmol each *ToxR*2-F3 and *ToxR*2-B3 primers, 1.5 mM MgCl_2_ and 1.25 U of *Taq* DNA polymerase (Promega, USA). The PCR reaction protocol involved an initial denaturation time of 3 min at 95 °C, followed by 40 cycles of amplification at 95 °C for 30 s, 56 °C for 30 s and 72 °C for 30 s, with a final extension step at 72 °C for 5 min. The PCR product was then purified using a PCR purification Kit (Qiagen, Germantown, MD, USA).

### 2.4. Recombinant Plasmid Construction for PCR and LAMP Analyses

The PCR amplicon (which was the target sequence for PCR and LAMP) was ligated into the plasmid pGEM-T easy vector (Promega, Madison, WI, USA) according to the manufacturer’s protocol. Next, the recombinant plasmid was transformed into 100 µL of *E. coli* strain JM109, followed by blue–white colony selection. Positive colonies indicated by a white color were collected and cultured for 16 h in an LB broth medium containing 100 µg ml^−1^ of ampicillin. Then, the plasmid extraction was performed using the PureYield ^TM^ Plasmid Miniprep System (Promega, Madison, WI, USA) according to the manufacturer’s protocol. The plasmid was then electrophoresed on a 1.5% agarose gel and purified for subsequent downstream processes.

### 2.5. Optimization of LAMP Reaction Condition with UV Analysis

The LAMP reactions were performed in a 25 µL reaction mixture containing 32 pmol each of the inner primers *ToxR*2-FIP and *ToxR*2-BIP, 8 pmol each of the outer primers *ToxR*2-F3 and *ToxR*2-B3, 32 pmol of the loop primers *ToxR*2-LB and *ToxR*2-LF, 1.4 mM deoxyribonucleotide triphosphate (dNTPs), 0.8 M betaine (Sigma-Aldrich), 20 mM Tris-HCl, 10 mM KCl, 10 mM (NH_4_)_2_SO_4_, 8 mM MgSO_4_, 0.1% Tween-20, 8 U *Bst* DNA Polymerase (NEB) and 5 µL of DNA sample. The LAMP reaction was performed on a heating block at 65 °C. The reaction was performed at 30, 40, 50 and 60 min for the reaction mixture without loop primers, and at 5, 10, 15 and 20 min for the reaction mixture with loop primers. The final products of the LAMP reaction were analyzed by 1.5% agarose gel electrophoresis.

### 2.6. Visualization of Amplification Products for LAMP Reaction Using SYBR Green

The LAMP reaction was carried out at 65 °C for 15 min. The resulting amplicons were electrophoresed using a 1.5% agarose gel, stained with ethidium bromide and visualized under UV light. For visualization using SYBR Green, approximately 2 µL of 1:10 diluted SYBR Green I nucleic acid gel stain was added directly into all reaction tubes containing the remaining LAMP-amplification products, and any changes of color was visually observed.

### 2.7. Sensitivity Test for the Detection of V. harveyi Using PCR and UV Analyses

A set of a 10-fold serial dilution of *V. harveyi* recombinant plasmid DNA samples was prepared from 60 ng to 0.6 fg. The PCR reactions were performed for all *V. harveyi* recombinant plasmid DNA samples using the *ToxR*2-F3 and *ToxR*2-B3 primers, yielding a PCR amplicon of approximately 245 bp. The amplification products were detected by 1.5% agarose gel electrophoresis followed by ethidium bromide staining and visualization by a UV transilluminator.

### 2.8. Sensitivity Test for the Detection of V. harveyi Using LAMP-UV Analysis and LAMP-SYBR Green

The same set of 10-fold serial dilutions of the *V. harveyi* recombinant plasmid DNA samples (60 ng to 0.6 fg) was used for the sensitivity test of the LAMP. The amplification products were then visualized in two ways; UV analysis and SYBR Green dye. The LAMP reaction was performed for all *V. harveyi* recombinant plasmid DNA samples at 65 °C for 15 min. The resulting amplicon of the LAMP reactions which were stained with ethidium bromide was analyzed on 1.5% agarose gel electrophoresis with the aid of a gel-documentation system. For visualization using SYBR Green dye, approximately 2 µL of 1:10 diluted SYBR Green I nucleic acid gel stain was added directly to all reaction tubes containing LAMP product amplicons, and any changes of color were visually observed.

### 2.9. Specificity Test of LAMP with UV Analysis for the Detection of Vibrio harveyi

In order to ensure that the developed procedure was specific to *V. harveyi*, the reference strains of the *Vibrio* and non-*Vibrio* bacterial samples were included in a specificity evaluation of the LAMP assays. These strains were *Vibrio parahaemolyticus* ATCC 17802, *Vibrio alginolyticus* ATCC 17749, *Vibrio aguillarum* ATCC 19264 and *Aeromonas salmonicida* subsp. *salmonicida* ATCC strain 33658. DNA templates were isolated from the bacterial cultures as described above for *V. harveyi,* according to the manufacturer’s procedures. The LAMP assay was carried out at 65 °C for 15 min.

### 2.10. Evaluation of LAMP-SYBR Green Assay with Grouper Infected with Vibrio harveyi

For the field evaluation, cell suspensions of *V. harveyi* in PBS were prepared prior to inoculation into healthy tiger groupers (*Epinephelus fuscoguttatus*). Initially, glycerol stock of *V. harveyi* was cultured in tryptic soy agar (TSA) supplemented with 2% NaCl at 30 °C overnight. Next, 1–2 mL of the cultured bacteria cells was transferred into a 2 mL microcentrifuge tube. Then, the tube was centrifuged for 1 min at 13,000 rpm, and the supernatant was discarded. To wash the cell, the pellet was re-suspended using PBS, centrifuged for 1 min at 13,000 rpm and the supernatant was discarded. This step was repeated three times. Finally, the pellet was re-suspended with approximately 1 mL of PBS. Three groupers were injected intraperitoneally with 10^4^ c.f.u/g of the *V. harveyi* PBS cell suspension. After the mortalities among the test group, we performed rapid detection using LAMP-SYBR green. A control group was included for comparison purposes. Fish from both the treated and control groups were dissected, and the internal tissues were used for the subsequent preparation of the DNA template using the rapid-boiling method [46]. Briefly, 200 µL of 0.025 N sodium hydroxide (NaOH) and 0.0125% sodium dedocyl sulphate (SDS) was added to the tissues and allowed to boil for 5 min. Approximately 200 µL of the supernatant solution was used directly for the subsequent LAMP-SYBR Green assay.

## 3. Results

### 3.1. LAMP Reaction Condition

**Figure 2:**
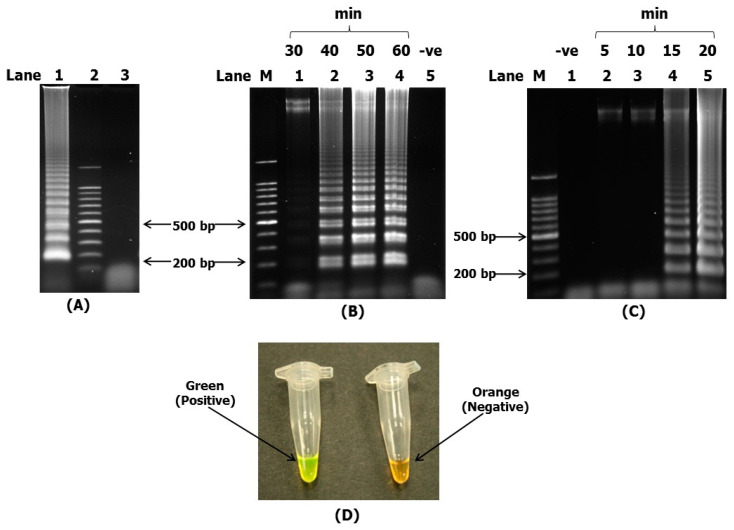
Gel electrophoresis of the LAMP assay and visualization with SYBR Green (**A**) An optimized LAMP protocol was developed for subsequent downstream tests, lane 1: *V. harveyi (*genomic DNA), lane 2: 100 bp DNA ladder, lane 3: negative control (distilled water). (**B**) Reaction mixture without loop primers, lane M: 100 bp DNA ladder, lane 5: negative control (distilled water); the reaction time of LAMP is indicated on the top of each lane. (**C**) Reaction mixture with loop primers, lane M: 100 bp DNA ladder, lane 1: negative control (distilled water); the reaction time of LAMP is indicated on the top of each lane. All products were analyzed by electrophoresis on a 1.5% agarose gel and stained with ethidium bromide. (**D**) Visual appearance of LAMP coupled with SYBR Green. Positive sample (green) and negative sample (orange).

The LAMP assay was performed to enable the quick identification of *V. harveyi.* For this purpose, we initially performed LAMP with just the four inner and outer primers, without the addition of the loop primers. However, we found that the best result was obtained with the addition of the loop primers. The loop primers used here consisted of a forward loop primer (ToxR2-LF) and a backward loop primer (ToxR2-LB). A ladder-like band was obtained after the LAMP amplicons were electrophoresed, stained with ethidium bromide and visualized under UV light. The ladder-like band was indicative of a successful LAMP reaction (Figure 2A).

All LAMP reactions were performed on a heating block at 65 °C. For comparison purposes, the assay was conducted at 30, 40, 50 and 60 min without loop primers, and 5, 10, 15 and 20 min for the reaction mixture with loop primers. The different interval periods were due to the time taken to obtain the ladder-like bands. The LAMP reaction without loop primers was able to detect *V. harveyi* (producing ladder-like bands) after about 40 to 60 min (Figure 2B). However, the LAMP reactions with loop primers only required about 10 to 15 min to detect *V. harveyi* (Figure 2C). Loop primers were used in all subsequent procedures throughout this study due to the fact that the reaction time was reduced by more than half compared with the LAMP reaction without loop primers. The primers hybridized to the loop region of the stem-loop DNA structure before initiating strand-displacement DNA synthesis. DNA synthesized from the loop primers was displaced by the extension from the 3′ end of the target DNA, thus generating structures which were not present in the conventional LAMP [26]. This enabled the drastic amplification and reduced the reaction time by more than half of that of the original LAMP reaction.

### 3.2. Visual Detection of Amplification Products of LAMP by SYBR Green

LAMP amplicons can be detected visually with naked eye either through turbidity or color changes by intercalating dye or fluorescent-labeled primers [47,48,49]. The addition of the SYBR Green I nucleic acid gel stain resulted in a color change from orange to green for *V. harveyi toxR* gene-positive reactions (Figure 2D), while negative reactions remained orange in color. We chose to use SYBR Green for easier visualization of the color changes in the field by less technically trained personnel. The use of the LAMP reaction also eliminated the need of gel electrophoresis with a gel-documentation system or a turbidimeter in the field.

### 3.3. Sensitivity Test of PCR-UV Analysis, LAMP-UV Analysis and LAMP-SYBR Green for the Detection of V. harveyi

**Figure 3:**
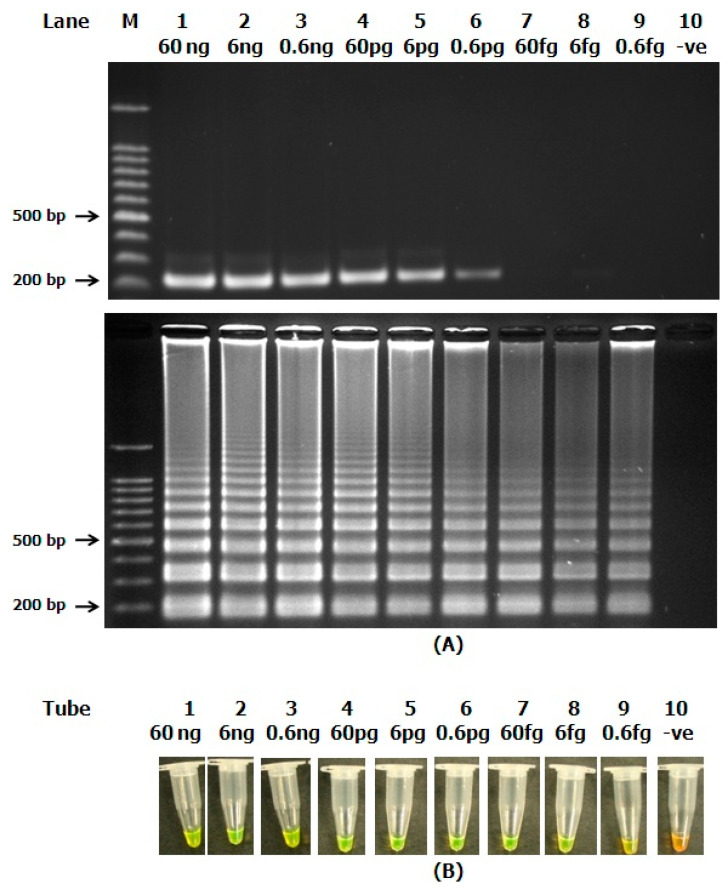
Results of sensitivity tests using PCR, LAMP-UV and LAMP-SYBR Green using different concentrations of recombinant plasmid of *V. harveyi* from 60 ng to 0.6 fg. End-point PCR (**A**, top), LAMP-UV analysis (**A**, bottom) and LAMP-SYBR Green (**B**). The concentrations of samples are indicated on the top of each lane and tube. Lane M: 100 bp DNA ladder, Lane 10: negative control (distilled water).

The sensitivity detection of the PCR assay was performed using a set of *V. harveyi* recombinant plasmid samples with different concentrations from 60 ng to 0.6 fg. The PCR reaction was performed using *ToxR*2-F3 and *ToxR*2-B3 primers. Both primers acted as the outer forward and reverse primers that bound at specific flanking regions, yielding a PCR amplicon of approximately 245 bp. The sensitivity of PCR was at 0.6 pg (Figure 3A, top).

The sensitivity level for the LAMP assay was also successfully tested using the same set of *V. harveyi* recombinant plasmid samples with the DNA plasmid concentration of 60 ng to 0.6 fg. The LAMP reaction was performed at 65 °C for 15 min using the optimized protocol, as described in the methodology section. The sensitivity test of the LAMP assay shows that the method is able to detect even as low as 0.6 fg of *V. harveyi* recombinant plasmid samples (Figure 3A, bottom).

The sensitivity tests of LAMP and SYBR Green were successfully conducted using the same *V. harveyi* recombinant plasmid samples from 60 ng to 0.6 fg, and the assay was also able to detect the *V. harveyi* recombinant plasmid samples at 0.6 fg (Figure 3B).

### 3.4. Specificity Test of LAMP and UV Analysis

**Figure 4:**
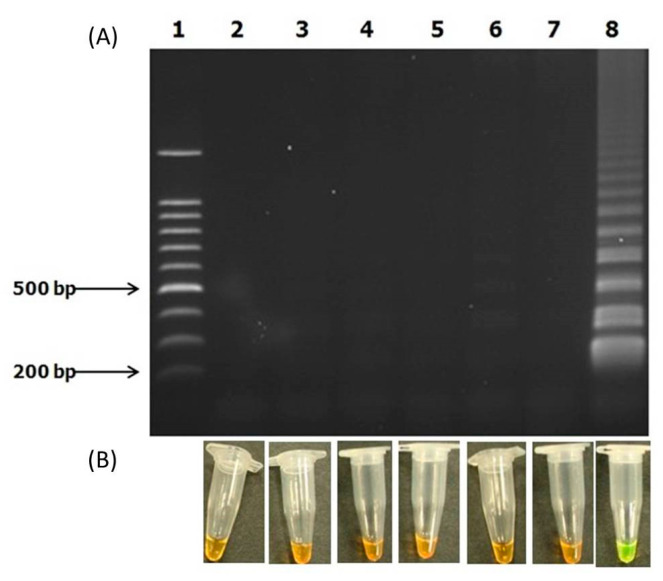
Specificity test for LAMP-SYBR green indicating the presence of amplicons for only *V. harveyi*. (**A**) LAMP amplicons visualized with gel electrophoresis in a 1.5% agarose gel and stained with ethidium bromide. (**B**) The corresponding LAMP assay visualized with SYBR green. Lane 1: 100 bp DNA ladder, Lanes 2–3: negative controls (distilled water), Lane 4: *Aeromonas salmonicides* subsp. *salmonicida* ATCC strain 33658, Lane 5: *V. aguillarium* ATCC 19264, Lane 6: *V. alginolyticus* ATCC 17749, Lane 7: *V. parahaemolyticus* ATCC 17802, Lane 8: *V. harveyi* strain VHJR 7.

The specificity test was successfully performed with the genomic DNA of the reference species as negative controls i.e., *Vibrio parahaemolyticus*, *V. alginolyticus*, *V. aguillarum* and *Aeromonas salmonicida* sub sp. *salmonicida*. We observed positive test results of ladder-like bands with only the *V. harveyi toxR* gene (Figure 4). All of the other species showed negative test results. The results for the specificity test show that the designed LAMP assay is specific to the *V. harveyi toxR* gene only, due to the fact that there were not any other positive results observed.

When designing the LAMP primers, we took care to avoid using conserved regions which were mutual with other *Vibrio* or closely related species. A BLAST search result of the target sequence shows that it is specific and unique to the reported strains of *V. harveyi*. The design of the primers was meticulously carried out in order to capture the various *V. harveyi* reported strains. The large capture pool (39 strains of *V. harveyi* as reported in GenBank) enabled the reduction of false negatives (Appendix A Table A1).

### 3.5. In Vivo Evaluation of Infected Grouper Using LAMP-SYBR Green

**Figure 5:**
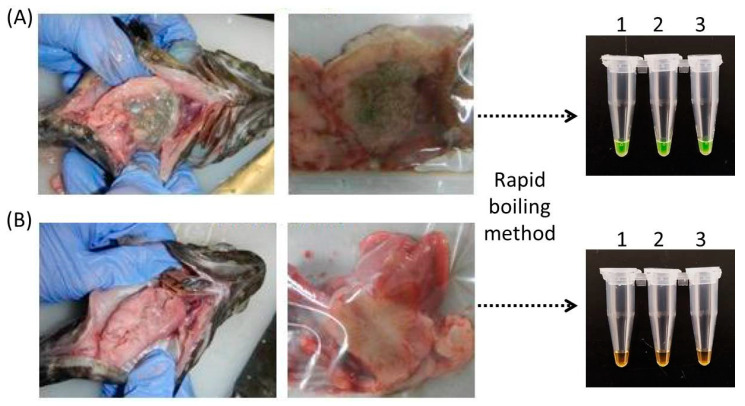
Evaluation of the improved LAMP-SYBR Green protocol on *Vibrio harveyi* infected grouper. (**A**) Sample of infected fish. (**B**) Sample of control non-infected fish. Tissue samples were obtained after 48 hrs of treatment, and the DNA template was prepared using the rapid-boiling method. The corresponding LAMP assay showed as being positive (green) for all three replicates, while the controls were negative (orange).

We infected groupers with a cell suspension of the *V. harveyi strain* VHJR7 and evaluated whether the newly designed LAMP primers could detect the presence of the pathogen in the fish. We used the rapid-boiling method used to avoid a lengthy DNA-isolation procedure. This took an additional 5 min on top of the 15 min required for LAMP amplification. The results indicate that the rapid-boiling method coupled with the newly designed primers was able to rapidly detect the presence of *V. harveyi*. Consequently, we could not detect the presence of *V. harveyi* in the control uninfected fish samples (Figure 5).

## 4. Discussion

A rapid, high-throughput yet simple and cost-effective detection method is needed to detect for the presence of *V. harveyi*. With this in mind, we optimized a procedure for the rapid detection of the pathogen by using the LAMP assay with the addition of two loop primers and a re-examination of the *toxR* gene sequences available at GenBank (www.ncbi.nlm.nih.gov (accessed on 1 February 2020)).

For successful LAMP amplification, the target region and design of the primers should be given considerable attention. A search conducted in GenBank resulted in more hits for the *toxR* genes compared with the other two genes, *dnaJ* and *VhhP2*, which were also reported to be used in LAMP assay for *V. harveyi* [38,39,40]. Hence, in order to capture as much sequence information as possible and translate them into a usable output, we decided to stick to the *toxR* gene initially used by Cao et al. [38]. However, we redesigned the primers to target conserved regions, taking into consideration all available sequence data in GenBank. With the limited complete genomes available in GenBank [50], selecting conserved regions for designing the LAMP primers was difficult. We took cognizant that *V. harveyi* strains are heterogeneous, as pointed out in several studies [51,52], which could impact the utility of the LAMP primers. Any mutations at the primer-binding regions of the various global strains would provide false negatives in the LAMP or PCR assays. Thus, we carefully screened all 39 available *toxR V. harveyi* sequences at GenBank.

To start off, we downloaded published sequences of the *Vibrio harveyi toxR* gene from GenBank, along with the initial reference sequence DQ640259.1. A blast search was performed to capture the conserved regions of the sequences and avoid nucleotide variants in the *V. harveyi* strains (Appendix A Table A1). After a manual search, we managed to identify a conserved target region with a length of approximately 246 bp. A set of six primers for LAMP was then designed according to the published sequence of the *toxR* gene of *V. harveyi* (GenBank ID: DQ640259.1). The set of six primers included the forward inner primer (ToxR2-FIP), backward inner primer (ToxR2-BIP), forward outer primer (ToxR2-F3), backward outer primer (ToxR2-B3), forward loop primer (ToxR2-LF) and backward loop primer (ToxR2-LB) (Figure 1). Primer Explorer (website link given in the methodology), which is a primer-designing software for LAMP, was used for this purpose. Two of the outer primers (ToxR2-F3 and ToxR2-B3) were initially used to perform PCR for the purpose of generating copies of the *V. harveyi* DNA amplicon to be used as templates for LAMP, in line with the approach used by others [46,53].

From our observations, the design of the LAMP primers for the *toxR* gene should be given special attention to prevent no or false amplification. In this regard, four key factors are important and need to be considered. These are the melting temperature (Tm), GC content, stability at the end of each primer and the formation of secondary structures. The optimal melting temperature (Tm) for each flanking region in this research was designed to range between 64 and 66 °C for F1c and B1c and between 59 and 61 °C for F2, B2, F3 and B3 to ensure appropriate primer-template complementarity for the generation of the ‘dumbbell’-like structure, which is unique to LAMP. In addition, the initiation of template amplification is highly sensitive to the ends of the primer sequences, and should contain a certain degree of stability. We ensured that the 3′ ends of the ToxR2-F2 or ToxR2-B2, ToxR2-F3 or ToxR2-B3 and the 5′ ends of ToxR2-F1c or ToxR2-B1c were designed so that the free energy was –4 kcal/mol or less for effective amplifications. The 5′ end of the ToxR2-F1c after amplification corresponded to the 3′ end of the ToxR2-F1. As such, stability is important as the reaction proceeds toward a negative change in free energy for effective LAMP reactions. Accordingly, due to the fact that the annealing reaction between the primer and the target DNA is an equilibrium reaction, it should proceed with a smaller change in free energy [54].

On another note, the GC contents of the inner and outer primer of our newly designed primers were between 50–61%, which was within the optimal range for effective amplification, i.e., 40% to 65% [54]. In addition, it is also important to note that the inner LAMP primers were designed so that they would not form secondary structures. The 3′ ends of the primers should be designed without complementary sequences to prevent the formation of primer dimers, which could interfere with the amplification.

A forward loop primer (ToxR2-LF) and backward loop primer (ToxR2-LB) are not essential requirements for LAMP. However, we included the loop primers as they managed to speed up the reaction, producing a rapid amplification of the LAMP products. With regards to time, the addition of loop primers resulted in the detection of LAMP products within 10–15 min, which was faster compared with the previously reported work on *V. harveyi* by Cao et al. [38] of approximately 60 min without the addition of loop primers. Furthermore, loop primers increase the specificity of the reaction because they require the recognition of eight distinct target sequences to form the LAMP structure needed for positive amplification [43]. Thus, they confer a higher amplification specificity compared with previous LAMP reactions without loop primers, which used only six distinct targets for the detection of *V. harveyi* [38,39,41]. In other pathogens, the addition of loop primers also resulted in a reduction of the detection time to 20 min for an infectious spleen and kidney necrosis virus (ISKNV) [55], 30 min for a hematopoietic necrosis virus (IHHNV) [46] and 30 min for a citrus bacterial cancer (CBC) [56].

From the sensitivity test, the detection limit of PCR was lower than the LAMP reaction. The sensitivity of PCR was only at 0.6 pg, while LAMP was able to detect lower amounts of the DNA sample, even at 0.6 fg (Figure 3A). We also observed a marked improvement in terms of sensitivity when using LAMP compared with end-point PCR assay [38].

With regards to the specificity test, the LAMP assay correctly managed only to detect the *V. harveyi toxR* gene and none of the controls, i.e., three *Vibrio* species (*V. aguillarium*, *V. alginolyticus* and *V. parahaemolyticus)* and one non-*Vibrio* (*Aeromonas salmonicides* subsp. *salmonicida*) strain (Figure 4). However, we do take note of the limited number of controls used in the specificity test, as we only used what was available to us at hand. A more-thorough screening will be needed for further confirmation.

The ability of SYBR Green to visualize the positive detection of *V. harveyi* using the naked eye through a color change, without the need for gel electrophoresis and a gel-documentation system, allows for it to be applied for a non-digital assay. Others have used different visualization dyes which could work as well, though with varying degrees of success, such as Calcein [57], PicoGreen [58], hydroxynaphthol blue dye [59], malachite green [60] or GelRed [61].

We proceeded to perform a test to detect the presence of *V. harveyi* in groupers inoculated with the pathogen. The boiling method seems to work well in generating rapid DNA templates for the LAMP assay. The color difference between orange to green was evident in the control and infected fish. However, we need to point out that while the data seems encouraging, we would need to test different tissue samples and with varying inoculation doses. There is a need to test the efficacy of the protocol and its limits in field trials, which were not conducted here.

Others working on LAMP-SYBR Green have pointed out that it may be difficult to differentiate between intermediate colors when low bacterial copies are present [22,23]. Thus, the need to move away from visual inspection to digitization could improve the reliability of the technique. One way to do this would be to use a turbidity meter, as this would be the cheapest and most cost-effective approach to obtain numerical measurements for cut-off purposes [62]. The use of a biosensor device coupled with LAMP would be another approach that could be employed [63,64]. For example, the integration of microfluidic chips with a real-time LAMP assay (dual-sample on-chip LAMP) can provide the simultaneous detection of multiple pathogens [65]. A more unconventional approach than that was the use of a nuclear-magnetic-resonance (NMR)-based biosensor approach utilizing iron nanoparticles coated with target-specific biomarkers for the rapid detection of a closely related *Vibrio* species, i.e., *V. parahaemolyticus* [66]. While these approaches are rapid and can minimize human errors (due to the manual visualization of color changes), one would need to consider the high cost to be borne by fish farmers.

Another factor to consider is that while we may be able to detect low copies of *V. harveyi* with LAMP or qPCR, this may not necessarily indicate the presence of the disease. The pathogen should be present in a sufficient amount in the environment targeting a susceptible host to trigger an infection. The body has innate and/or adaptive immune responses to deal with nascent contact. This has led to other methods to detect *V. harveyi* infection based on the response signals of host species. One such approach is to use small, non-coding RNA molecules such as microRNA (miRNA) [67,68] and Piwi-interacting RNA (piRNA) [69,70] obtained from the extracellular vesicles of skin mucus. The up or downregulations of the expressed piRNA and miRNA genes can be used as biomarkers to predict the onset of the diseases. Alternatively, certain protein biomarkers such as Ferritin, toll-like receptor 5S protein and calcium-transporting ATPase, Histone H2B and Eukaryotic translation-initiation factor 5A have been implicated in the disease’s formation [71]. These novel, non-invasive approaches have been shown to be able to differentiate between diseased and healthy fish (*Cynoglossus semilaevis*). Incorporating the miRNA and piRNA biomarkers with LAMP and integrating it with a rapid-visualization approach may have practical value for large-scale disease screening. The downside of this is that the expression profile of the miRNA, piRNA and protein biomarkers must be consistently expressed across all marine species to be effectively used in the wide host range of the *V. harveyi* infection.

With regards to future developments, we proceeded to incorporate LAMP in conjunction with lateral-flow dipstick (LFD) for *V. harveyi* and to perform more comprehensive field trials, which will be presented separately. We feel that researchers could benefit from the LAMP-SYBR Green procedure used here as it is straightforward and without the need for much optimization.

## 5. Conclusions

The development of the LAMP-SYBR Green assay will provide a valuable tool for the rapid detection of the *V. harveyi* infection, both in laboratories and especially in the field. While we acknowledge the need to conduct field trials, we thought that it would be best to put out the LAMP primers and protocols for researchers to start testing them, as it would be impossible to obtain all strains of *V. harveyi* for screening. The assay can be adapted easily for the rapid surveillance and detection of *V. harveyi* in fish and other aquatic animals in developing countries with low operation expenditures and without the need for costly equipment or skilled personnel.

## Figures and Tables

**Table 1:** The sequences and lengths of the inner, outer and loop primers based on the *toxR* gene of *V. harveyi.* The primers were designed using the default parameters of Primer Explorer Version 4 at http://primerexplorer.jp/elamp4.0.0/index.html (accessed on 1 February 2020).

Name	Sequence (5′-3′)	Length (bp)
*ToxR*2-FIP	ATGGTTGAGCTGTCGGTGCTTGTTTCAGAGCCCACTGCTGAGA	43
*ToxR*2-BIP	TCATCGTGTTAGTTGCCCTGCTTTTTTGTGATTCTGCAGGGTTGG	45
*ToxR*2-F3	GAAGCTCCAGTCGTTGACTT	20
*ToxR*2-B3	CATCACTGGCACGTTGTGA	19
*ToxR*2-LF	TCGACGGCTGTTTCTGCTTTT	21
*ToxR*2-LB	TCCTGTTGGCGTGTTAATGCT	21

## Data Availability

Not applicable.

## Appendix A

**Table A1:** BLAST search for the specific target sequence of *Vibrio harveyi* based on the *toxR* gene. The BLAST search produced a hit of 39 strains of the *V. harveyi toxR* gene (Parameters: nucleotide collection (nr/nt) and highly similar sequence).

No	Sequence Description	Identities	Max identical	Accession Number
1	*V. harveyi* strain ATCC 35084	245/245	100%	JF930599
2	*V. harveyi* strain VIB 645	245/245	100%	DQ640259
3	*V. harveyi* strain CAIM 363	245/245	100%	DQ640256
4	*V. harveyi* strain CAIM 79	245/245	100%	DQ640255
5	*V. harveyi* strain CAIM 520	245/245	100%	DQ517446
6	*V. harveyi* strain 7207	244/245	99%	JF930600
7	*V. harveyi* strain LPD 1-3-12	244/245	99%	FR719019
8	*V. harveyi* strain VIB 572	244/245	99%	DQ640258
9	*V. harveyi* strain CAIM 1792	244/245	99%	DQ640257
10	*V. harveyi* strain M028	243/245	99%	JF930597
11	*V. harveyi* strain ATCC 43516	243/245	99%	JF930595
12	*V. harveyi* strain ATCC 33868	243/245	99%	JF930596
13	*V. harveyi* strain LMG 4044	243/245	99%	JF930594
14	*V. harveyi* strain LPD 1-3-31	243/245	99%	FR719020
15	*V. harveyi* strain LPD 1-3-11	243/245	99%	FR719018
16	*V. harveyi* strain LPD 1-3-10	243/245	99%	FR719017
17	*V. harveyi* strain LPD 1-1-79	243/245	99%	FR719015
18	*V. harveyi* strain LPD 1-1-39	243/245	99%	FR719014
19	*V. harveyi* strain SA F3s 55	243/245	99%	FR719011
20	*V. harveyi* strain SA F3s 38	243/245	99%	FR719010
21	*V. harveyi* strain DPD 4-1-7	243/245	99%	FR719009
22	*V. harveyi* strain LPD 1-3-35	243/245	99%	FM202687
23	*V. harveyi* strain LPD 1-3-27	243/245	99%	FM202686
24	*V. harveyi* strain LPD 1-1-10	243/245	99%	FM202684
25	*V. harveyi* strain LPD 1-3-1	243/245	99%	FM202685
26	*V. harveyi* strain DL F5 57	243/245	99%	FM202681
27	*V. harveyi* strain H2	243/245	99%	FM202680
28	*V. harveyi* strain SA F3s 40	243/245	99%	FM202679
29	*V. harveyi* strain culture collection	243/245	99%	FM202678
30	*V. harveyi* strain VIB 658	243/245	99%	DQ640261
31	*V. harveyi* strain VIB 653	243/245	99%	DQ640260
32	*V. harveyi* strain CAIM 512	243/259	99%	DQ503438
33	*V. harveyi* strain CAIM NBRC 15634	243/259	99%	DQ403146
34	*V. harveyi ToxR* (*toxR*)	243/245	99%	AY247418
35	*V. harveyi* strain C259	242/245	99%	JF930598
36	*V. harveyi* strain LPD 1-1-7	242/245	99%	FR719012
37	*V. harveyi* strain SA F 4s 17	241/245	98%	FM202683
38	*V. harveyi* strain SA F 1s 10	241/245	98%	FM202682
39	*V. harveyi* strain H050704-1	241/245	98%	EF645830
